# Toward an Occupational Justice–Informed Vocational Rehabilitation Framework for Mental Health Service Users in Namibia: A Delphi Study

**DOI:** 10.1155/oti/7144809

**Published:** 2026-11-03

**Authors:** Munyaradzi Chimara, Hester van Biljon, Fasloen Adams, Lana van Niekerk

**Affiliations:** ^1^ Division of Occupational Therapy Faculty of Medicine and Health Sciences Stellenbosch University Cape Town South Africa sun.ac.za; ^2^ Department of Occupational Therapy and Physiotherapy School of Allied Health Sciences Faculty of Health Sciences and Veterinary Medicine University of Namibia Windhoek Namibia unam.na

**Keywords:** disability policies, good health and well-being, mental health service users, occupational justice, place-and-train model, work readiness

## Abstract

**Introduction and Rationale:**

A vocational rehabilitation practice framework is needed to promote shared clinical reasoning among occupational therapists and support consistent, contextually relevant services for mental health service users in Namibia. This study sought expert consensus on foundational components that could guide the future development of such a framework. An occupational justice orientation informed the conceptual framing, viewing vocational rehabilitation as both a therapeutic and advocacy‐based process aimed at promoting equitable access to meaningful work.

**Methods:**

A modified Delphi method was employed to obtain consensus from a panel of 11 experts on 50 statements derived from three datasets. The process included two Delphi survey rounds, conducted using a 3‐point Likert scale via REDCap software. Consensus was defined as 75% or greater agreement on a given item.

**Results:**

Consensus was reached on 38 of the 50 statements (76%). Items endorsed by the panel emphasized foundational strategies, including advancing occupational justice through advocacy for equitable work opportunities, engaging families and employers, applying individualized and client‐centered approaches such as place‐and‐train, and aligning services with national mental health and disability policies. These findings represent areas of shared professional priority rather than a finalized practice framework.

**Conclusion:**

The study demonstrates consensus among experts that vocational rehabilitation practice in Namibia requires attention to both individual work‐readiness support and the broader systemic conditions that shape access to meaningful work for service users. The priorities identified offer a critical evidence base that will inform and shape the final phase of developing the vocational rehabilitation framework.

## 1. Introduction

Occupational therapists play a vital role in the vocational rehabilitation of mental health service users. In the Namibian context, vocational rehabilitation for mental health service users remains a challenge because of factors such as limited vocational rehabilitation resources and services, lack of a clear vocational rehabilitation strategy, and a general lack of mental illness awareness [1]. A need to develop a contextually relevant occupational therapy vocational rehabilitation practice framework was identified to guide Namibian occupational therapists when providing such services at hospital level. This need became apparent to the first author as he worked in Namibia, as well as with occupational therapists in mental health institutions. The absence of a practice framework to guide occupational therapists in providing vocational rehabilitation for service users with chronic mental illness had led to uncertainty and inconsistency in intervention planning and implementation. This posed a challenge particularly to junior occupational therapists who view vocational rehabilitation as a specialized area that is not given adequate attention at undergraduate level [2]. This identified need prompted the development of a contextually relevant Namibian vocational rehabilitation practice framework to move toward ensuring a common reference point among therapists, consistency across settings, and appropriateness of occupational therapists′ vocational rehabilitation services [1].

Several vocational rehabilitation models have been developed globally to support mental health service users in accessing and sustaining work or employment. These include the Individual Placement and Support (IPS) model, supported employment, the clubhouse model, the traditional vocational rehabilitation (train‐and‐place) approach, and the vocational empowerment model among others [3]. However, these models have limited applicability within the Namibian context for several reasons. First, Namibia′s high unemployment rate and significant barriers to competitive employment make the emphasis on rapid placement into competitive jobs largely unrealistic for many service users [4]. Second, these models are predominantly grounded in Eurocentric and individualistic perspectives, which often overlook collectivist African values, communal support systems, and indigenous understanding of work and productivity. Consequently, they fail to adequately address the sociocultural and structural realities that shape vocational participation in Namibia. Given these limitations, there was a need to develop a contextually responsive vocational rehabilitation practice framework that reflects Namibia′s unique socioeconomic conditions, cultural values, and system challenges.

A framework can be defined as “a system of rules, ideas, or beliefs that is used to plan or decide something” [5] (pg511), whereas in the context of this study, a practice framework is a conceptual map that brings together occupational therapists′ practice in vocational rehabilitation for chronic mentally ill service users thus providing a clear understanding of intervention in this particular field [6]. Connolly [6] asserts that a practice framework can also be utilized as a tool that offers a theory‐driven framework for intervention, accompanied by key prompts that facilitate optimal practice. The literature highlights a number of benefits of having a practice framework. These include standardization of practice, quality assurance, professional development, accountability, integration of knowledge, guidance in complex situations, collaboration and communication, client‐centered focus, resource allocation, and cultural competency and sensitivity [7].

A Delphi method is one of the most appropriate structured approaches to gaining expert consensus when developing a practice framework [8]. One advantage of a Delphi method is that experts have the opportunity to agree or disagree without direct confrontation; hence, there is less likelihood of conforming with the dominant view [9]. The controlled feedback process is another advantage as it allows and encourages experts to reassess their initial judgments in the previous round thus enhancing validity of the data [9]. Experts are integral in the development of a vocational rehabilitation practice framework in a number of ways. Their clinical experience in the practice context and understanding of the service users ensures the framework is relevant and applicable in the setting. On the other hand, experts from the academic field are usually well‐versed in the current evidence, putting them in a stronger position to assess and provide plausible recommendations. Therefore, it is justifiable to utilize a Delphi method to reach expert consensus on items that should be included in an occupational therapy vocational rehabilitation practice framework.

This study, and indeed the whole PhD project for the first author, is grounded in an occupational justice framework to ensure contextual relevance in the Namibian setting. Recognizing work as a vital occupation that contributes to recovery and life satisfaction for mental health service users, the study applied concepts such as occupational deprivation, marginalization, and apartheid to examine structural and social barriers to equitable work participation [10]. These occupational justice concepts guided the analysis of how Namibian systems and contexts influence access to vocational opportunities, thus informing the development of a culturally responsive and socially just vocational rehabilitation practice framework. The development of the framework followed four phases. Phase 3, a Delphi study to obtain expert consensus on factors that should be considered for the framework, is reported in this article. Preceding phases were (i) a scoping review of vocational rehabilitation interventions in low‐income and middle‐income countries [3] and (ii) stakeholder engagement to explore their views on vocational rehabilitation in Namibia [1]. In the final phase, the findings from the aforementioned phases will be integrated into an occupational therapy vocational rehabilitation practice framework.

This Delphi study is therefore aimed at obtaining consensus from vocational rehabilitation experts on the items that should be included in an occupational therapy vocational rehabilitation practice framework for mental health service users in Namibia.

## 2. Methods

### 2.1. Design

A Delphi method was used to obtain consensus from a panel of experts in vocational rehabilitation from the southern Africa region. The experts were required to agree on the importance of each of the 50 statements (items) on vocational rehabilitation for mental health service users. The statements were drawn from three sources: (i) a scoping review of vocational rehabilitation for mental health service users in low‐income and middle‐income countries [3], (ii) perspectives of service providers on factors that should be included in a vocational rehabilitation framework for mental health service users in Namibia [1], and (iii) service users′ perspectives on factors to consider for vocational rehabilitation in Namibia [1, 3]. “Guidance on Conducting and REporting DElphi Studies (CREDES)” in conjunction with “Research Guidelines for the Delphi Survey Technique” were used for drafting and reporting this study [11] [8].

The CREDES guidelines helped shape the Delphi questions by making sure they were clear, fair, and purposeful. Also, the guidelines encouraged the research team to carefully review and refine each statement before sharing them with the experts. As a result, the final questions were easy to understand, avoided bias, and were structured in a way that allowed experts to give thoughtful ratings across the two rounds of the Delphi process.

### 2.2. Participants and Recruitment

The expert panel for this study comprised qualified occupational therapists who met any one of the following inclusion criteria: (i) a minimum of 2 years as service provider in a public mental health care setting in Namibia at the time of data collection; (ii) a minimum of 2 years working as a university lecturer for occupational therapy training program, with a specialization in teaching and research related to vocational rehabilitation; and (iii) a minimum of 2 years of working experience in a specialized vocational rehabilitation facility in any southern African country including Namibia. Although 5 years is often cited as a typical benchmark for expertise in Delphi studies, the present study accepted participants with minimum of 2 years of relevant experience because of limited expert pool in the specialized area of vocational rehabilitation and mental health in southern Africa [12, 13].

Authors followed a three‐step process to recruit the study participants. First, a worksheet was prepared to identify public mental health care institutions in Namibia, vocational rehabilitation institutions in southern African countries, and universities in southern African countries that train occupational therapists and provide specialization in vocational rehabilitation. Second, authors populated the worksheet with names of occupational therapists working in the identified institutions. Third, screening was conducted to determine eligibility according to the set criteria, followed by an email inviting the experts to participate in the study.

### 2.3. Procedure

Two Delphi rounds were conducted between January and April 2025 using REDCap software [14] as shown in Figure 1. The questionnaire for the first round had two sections. The first section had a total of 50 closed‐ended questions covering five themes from three sources mentioned earlier, whereas the second section comprised nine questions on the demographic profile of the experts. The questions were created by weaving together insights from all three sources. The scoping review [3] offered a broad picture of what vocational rehabilitation looks like in similar low‐income and middle‐income settings, whereas service providers [1] in Namibia contributed their practical understanding of what actually works on the ground. At the same time, service users [4] shared what they personally need and value in the vocational rehabilitation process. By comparing and blending these three perspectives, the research team shaped the 50 statements, so they reflected both evidence and real‐life experiences, ensuring the questions were meaningful, relevant, and grounded in the Namibian context.

**Figure 1:**
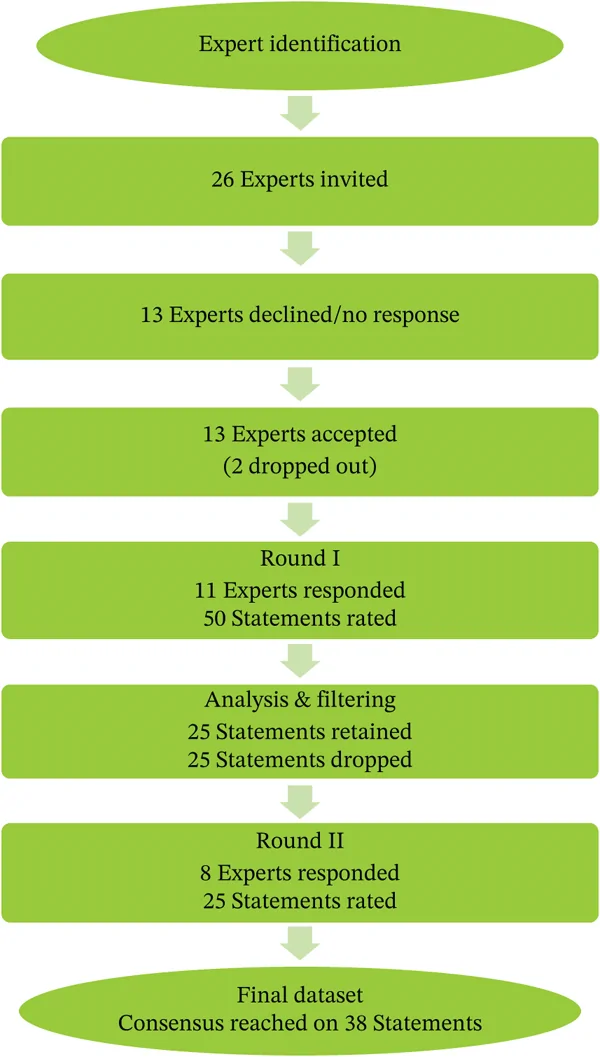
Delphi consensus process flow diagram. Summary of expert recruitment, participation, and statement selection across two Delphi rounds, resulting in consensus on 38 statements.

For closed‐ended questions, the experts were required to rate the level of importance of each item on a 3‐point Likert scale, which are *not important*, *important*, and *very important*. In addition to the closed‐ended questions, Round 2 included an optional open‐ended qualitative justification field, allowing experts to explain ratings, request clarity, or provide general comments. An email was sent to all 23 identified experts who met the inclusion criteria. The email provided information about the study including details of the researchers, aim of the study, and ethical clearance, as well as expectations from the researchers. Experts who gave consent to participate in the study followed a link to the survey questions. It took an average of 20 min to complete each survey.

### 2.4. First Round

A Microsoft Excel spreadsheet was utilized for analysis of data received in the first round. The frequency of rating for each statement in terms of level of importance was computed. Consensus was deemed reached if level of importance received at least 75% of the rating. Out of the 50 statements that were presented, consensus was reached on 25 statements, whereas the remaining 25 statements were sent through to second round of the Delphi as presented in Table 1.

**Table 1:** Results for first round and second round Delphi surveys.

No.	Item	Delphi Round I results				Delphi Round II results			
		Not important	Important	Very important	Consensus (75% agreement) reached?) Y/N	Not important	Important	Very important	Consensus (75% agreement reached?) Y/N
Collaborative advocacy to create equitable work opportunities for service users with chronic mental illness									
1.	Occupational therapists should educate family members of mental health service users to improve their mental health literacy.	0%	9%	91%	Yes				
2.	Occupational therapists should educate employers to improve their understanding of mental illness and the potential impact of mental illness on the worker.	0%	18%	82%	Yes				
3.	Occupational therapists should educate policy makers on the importance of vocational rehabilitation for mental health service users.	0%	27%	73%	No	0%	25%	75%	Yes
4.	Occupational therapists should educate members of the hospital management on vocational rehabilitation for mental health service users.	0%	45%	55%	No	0%	12.5%	87.5%	Yes
5.	Occupational therapists should educate traditional leaders on vocational rehabilitation of mental health service users.	0%	73%	27%	No	0%	62.5%	37.5%	No
6.	Occupational therapists should engage the multidisciplinary team in vocational rehabilitation.	0%	45%	55%	No	0%	12.5%	87.5%	Yes
7.	The multidisciplinary team engaged in vocational rehabilitation should ideally include occupational therapists, mental health nurse, social worker, medical officer, and clinical psychologist.	9%	28%	63%	No	0%	37.5%	62.5%	No
8.	Occupational therapists who are working for the Ministry of Health and Social services should facilitate stakeholder agreements for vocational rehabilitation for mental health service users.	0%	27%	73%	No	0%	37.5%	62.5%	No
9.	The Ministry of Health and Social services should facilitate stakeholder agreements for vocational rehabilitation for mental health service users.	9%	18%	73%	No	12.5%	25%	62.5%	No
10.	Family members should be included in vocational rehabilitation of a mental health service user.	0%	45%	55%	No	0%	25%	75%	Yes
11.	The employer community should be included in vocational rehabilitation of a mental health service user.	0%	27%	73%	No	0%	0%	100%	Yes
12.	Alcohol and substance use rehabilitation should be part and parcel of vocational rehabilitation for mental health service user.	0%	46%	64%	No	0%	50%	50%	No
13.	Occupational therapists should work on improving mental health service users′ emotional insight on the importance of taking medication in vocational rehabilitation.	0%	46%	64%	No	0%	50%	50%	No

The importance of promoting mental health service users′ rights to equitable work opportunities									
14.	Occupational therapists should advocate for mental health service users for their right to equitable work opportunities.	0%	18%	82%	Yes				
15.	Mental health service users should be given the opportunity to work regardless of the severity of mental illness.	27%	45%	27%	No	0%	75%	25%	Yes
16.	Mental health service users should receive wages and salaries that commensurate with work done.	9%	9%	82%	Yes				

Vocational rehabilitation resources and services									
17.	Occupational therapists should advocate for human resources to conduct vocational rehabilitation.	0%	27%	73%	No	0%	12.5%	87.5%	Yes
18.	Occupational therapists should receive regular training on vocational rehabilitation for mental health service users.	0%	46%	64%	No	0%	50%	50%	No
19.	Occupational therapists in state mental health facilities should budget for vocational rehabilitation.	0%	18%	82%	Yes				
20.	Occupational therapists in state mental health facilities should plan for vocational rehabilitation structures.	0%	18%	82%	Yes				

Vocational rehabilitation intervention strategy for service users with chronic mental illness									
21.	Occupational therapists should employ “place‐and‐train” strategy to vocational rehabilitation of mental health service users.	0%	18%	82%	Yes				
22.	Occupational therapists should negotiate with employers for support in vocational rehabilitation of mental health service users.	0%	9%	91%	Yes				
23.	Occupational therapists should negotiate for reasonable accommodation of mental health service users.	0%	9%	91%	Yes				
24.	Occupational therapists should conduct work visits to support mental health service users in the workplace.	0%	9%	91%	Yes				
25.	Vocational rehabilitation for mental health service users should include building skills and capacity for self‐employment.	0%	27%	73%	No	0%	25%	75%	Yes
26.	Occupational therapists should provide vocational guidance and counseling to mental health service users.	0%	27%	73%	No	0%	25%	75%	Yes
27.	Occupational therapists should identify placements for mental health service users to work such as sheltered workshops.	0%	27%	73%	No	0%	50%	50%	No
28.	Occupational therapists should teach mental health service users self‐management skills.	0%	18%	82%	Yes				
29.	Systematic involvement of family members and social networks to help with job seeking.	0%	18%	82%	Yes				
30.	Occupational therapists should facilitate work for mental health service users in mental health service users′ family businesses.	18%	27%	55%	No	12.5%	75%	12.5%	Yes
31.	Occupational therapists should conduct job analysis and job matching.	0%	0%	100%	Yes				
32.	Occupational therapists should do personal life skills coaching.	0%	9%	91%	Yes				
33.	Occupational therapists should train mental health service users basic self‐employment skills.	0%	9%	91%	Yes				

Vocational rehabilitation policy for service users with chronic mental illness									
34.	Vocational rehabilitation for mental health service users should be conducted in the community.	0%	46%	64%	No	0%	12.5%	87.5%	Yes
35.	Vocational rehabilitation for mental health service users should start in the hospital.	9%	36%	55%	No	0%	50%	50%	No
36.	Occupational therapists should facilitate setting up of employee assistance programs for mental health service users.	0%	45%	55%	No	0%	37.5%	62.5%	No
37.	Occupational therapists should utilize the National Policy on Disability to guide provision of vocational rehabilitation services.	0%	18%	82%	Yes				
38.	Occupational therapists should utilize the National Policy on Mental Health to guide the provision of vocational rehabilitation services.	0%	18%	82%	Yes				
39.	Occupational therapists should identify mental health service users who need vocational rehabilitation early.	0%	18%	82%	Yes				
40.	Vocational rehabilitation assessment of mental health service users by occupational therapists should happen as soon as they are admitted in hospital.	18%	36%	45%	No	0%	50%	50%	No
41.	Occupational therapists should apply case management as an intervention approach.	0%	46%	64%	No	0%	25%	75%	Yes
42.	Case management should involve developing a care plan and vocational training to aid work re‐entry.	0%	18%	82%	Yes				
43.	Occupational therapists should maintain follow‐up of mental health service users receiving vocational rehabilitation service.	0%	27%	73%	No	0%	25%	75%	Yes
44.	Occupational therapists should view vocational rehabilitation for mental health service users as a human rights issue.	0%	9%	91%	Yes				
45.	Vocational rehabilitation for mental health service users should be client‐centered, holistic, and culturally sensitive.	0%	9%	91%	Yes				
46.	Occupational therapists should ensure that vocational rehabilitation for mental health service users is cost‐effective.	9%	18%	73%	No	0%	12.5%	87.5%	Yes
47.	Occupational therapists should provide ongoing individualized support.	0%	18%	82%	Yes				
48.	Vocational rehabilitation should be planned with the client.	0%	18%	82%	Yes				
49.	Vocational rehabilitation intervention should optimize the fit between an individual′s abilities and the environmental demands.	0%	18%	82%	Yes				
50.	Occupational therapists should empower the mental health service users and their family.	0%	18%	82%	Yes				

### 2.5. Second Round

The second round Delphi questionnaire elicited qualitative data in addition to the quantitative data as the authors continued their pursuit on gaining expert consensus on factors that should be included in an occupational therapy vocational rehabilitation practice framework for chronic mentally ill service users. The participants had the opportunity to review the results from the first round. As in first round, the survey required experts to rate the importance of the statements regarding vocational rehabilitation. In addition, the experts had an option to qualitatively provide justification for their rating, provide a general comment, or to ask for clarity on the statement presented. Out of the 25 statements presented in the second round, expert consensus was reached on 13 statements (Table 1). The Delphi process was concluded after the second round as consensus was reached on 76% of the items, and participant responses showed little variation from the first round, indicating stability of expert opinion [15] [8]. This stability was confirmed by comparing how experts rated each item across the two rounds and finding that their views remained largely unchanged, showing that their judgments had settled, and additional rounds were unlikely to produce new insights.

## 3. Results

### 3.1. Demographics of the Participants

A total of 26 experts in vocational rehabilitation and mental health were invited to participate in the Delphi study, of whom 13 responded and 11 completed the first round and eight completed the second round. The panel was composed of qualified and experienced occupational therapists, with 73% (*n* = 8) holding at least a master′s degree and 18% (*n* = 2) holding a doctorate. Most participants **(**91%**)** had more than 6 years of experience practicing occupational therapy in an African country. Experience in vocational rehabilitation was more moderate, with 54% (*n* = 6) reporting 2–5 years in the field, whereas experience in mental health was more extensive, as seven participants had over 10 years in mental health practice. The professional roles of participants were diverse: 73% were educators, 45% clinicians, 36% researchers, and 9% administrators. Notably, no participants identified as policy makers, which possibly limited the policy perspective in the findings. Overall, the Delphi panel brought multifaceted expertise, especially in education, mental health, and clinical practice, providing strong grounding for the study′s focus on vocational rehabilitation for mental health service users in an African context. Table 2 below provides a summary of the demographic profile of the participants.

**Table 2:** Participant demographic information.

Participant demographic information		*N*	%
Highest level of education	Bachelor′s degree	3	27%
	Master′s degree	6	54%
	Doctorate degree	2	18%

Current job/employment field	Clinician	5	45%
	Educator	8	73%
	Policy maker	0	0%
	Administrator	1	9%
	Researcher	4	36%

Years of experience as an occupational therapist in Africa	2–5 years	1	9%
	6–10 years	3	27%
	11–15 years	3	27%
	16–20 years	1	9%
	More than 20 years	3	27%

Years of experience in vocational rehabilitation	2–5 years	6	54%
	6–10 years	2	18%
	11–15 years	0	0%
	16–20 years	1	9%
	More than 20 years	2	18%

Years of experience in mental health	2–5 years	2	18%
	6–10 years	2	18%
	11–15 years	3	27%
	16–20 years	3	27%
	More than 20 years	1	9%

Years of experience in occupational therapy education	2–5 years	2	18%
	6–10 years	4	36%
	11–15 years	2	18%
	16–20 years	0	0%
	More than 20 years	2	18%
	None	1	9%

### 3.2. Collaborative Advocacy to Create Work Opportunities

Thirteen items were presented to experts on the theme “collaborative advocacy to create work opportunities” as shown in Table 1. Consensus was reached on two items in the first round, whereas a further five items were agreed upon in the second round. The experts provided comments to support their rating of the statements. They emphasized that occupational therapists should support service users to enter the labor market. In addition, occupational therapists should educate employers, service users′ coworkers, family members, and the community at large on the importance of work to service users. Interdisciplinary collaboration and community buy‐in were seen as essential for effective vocational rehabilitation. Below is a quote from Expert 4:

As occupational therapists, it is our role to support people with disabilities to enter the labor market and contribute to society, fostering independence and meaningful engagement. In order to do this for our clients, it is important to also consider the context and environment in which they work, and educating employers, colleagues, family, the client and the community at large may often be necessary in order for clients to engage fully. This may be done in many different ways, such as educating people on the rights of persons with disabilities, on the importance of employment and support therein, assessing suitable work stations and placement for clients, and combating stigma and discrimination. Working with multiple stakeholders will aid this process and will make vocational rehabilitation more effective overall, as you need the buy‐in from the clients, yet also the community to make rehabilitation a success. (Expert 4)

### 3.3. Promoting MHSUs Rights to Equitable Work Opportunities

Three items were submitted to experts for rating under this theme. Experts reached consensus on all three items. The experts concurred that occupational therapists should advocate for service users for their rights to equitable work opportunities. To support this point, one expert noted that “every person has the right to work according to Article 27 of the Convention on the Rights of Persons with Disabilities.” The results further revealed that there should be a variety of work options for all levels of function. The severity of mental illness should be considered when advocating for work. It is important for occupational therapists to note that it can be stressful to link certain service users to work opportunities. Also, service users should receive wages and salaries that commensurate with the scope/level of their work.

### 3.4. Vocational Rehabilitation Resources and Services

Regarding vocational resources and services, four items were submitted to experts for consensus of which three items reached consensus. It was reported that lifelong learning for occupational therapists in vocational rehabilitation is important because the field is fluid requiring updates with the current trends in the labor market. Also, continuous professional development is a requirement for health care professions including occupational therapists. Another expert stated that there is an increase in the demand for vocational rehabilitation services, and there is a need to engage skilled artisans.

HR (human resource) practitioners play a crucial role in many aspects of voc rehab, but they also sometimes do not exist (e.g., in the case of small businesses). Where they exist, it′s very important that OTs advocate and involve them. With regards to regular training, OTs not specifically working in voc rehab may not need regular training as long as their basic qualifications equipped them with a broad understanding of voc rehab and when to refer clients to practitioners who focus on this. For voc rehab practitioners ourselves, lifelong learning is critical due to the fluid nature of economies and labor markets—including changing regulations relevant to clients with disabilities, disability definitions, equity requirements like headcounts, quotas and targets, reasonable accommodation options, what is considered to be unjustifiable hardship, dismissal procedures, etc., etc. (Expert 3)

### 3.5. Vocational Rehabilitation Interventions Strategy

Thirteen items were rated under the theme “vocational rehabilitation strategy” of which nine items reached consensus in first round and a further three items in the second round. Experts reported that all forms of employment should be explored for mental health service users, and occupational therapists should advocate for service users to enter work in the appropriate facility. Items under this theme include place‐and‐train strategy, reasonable accommodation, work visits, vocational guidance and counseling, social networks, and job analysis and coaching, as well as basic self‐employment skills.

The ability of an OT to identify protective factors and resources that could be utilized by the client to attain their vocational goal is key. Hence, all forms of employment (mainstream, sheltered, self‐employment, family or community integrated, etc.) should be explored. (Expert 6)

### 3.6. Vocational Rehabilitation Policy

Experts reached consensus in 14 out of 17 items presented to them under the theme “vocational rehabilitation policy.” Occupational therapists should utilize the National Policy on Disability and the National Policy on Mental Health to guide the provision of vocational rehabilitation services. Vocational rehabilitation should be client‐centered, holistic, and culturally sensitive. Planning should be done with the client and ongoing individualized support should be provided. The findings further revealed that starting vocational rehabilitation as soon as possible may not be appropriate because of individual factors such as social withdrawal, suicide ideation, or severe dysfunction. Community‐based vocational rehabilitation intervention received consensus, and one expert noted that community interventions facilitate social inclusion.

Early intervention is key for a better functional prognosis; hence, if interventions start during hospitalization, the likelihood of postdischarge engagement increases. Community‐based interventions facilitate social inclusion. Routine follow‐ups ensure outcome measuring as well as an opportunity to reassess and pivot interventions to meet changing needs. (Expert 6)

## 4. Discussion

### 4.1. Introduction

This study is aimed at measuring consensus of occupational therapists, who are experts in vocational rehabilitation, on items that should be included in a vocational rehabilitation practice framework. This framework is intended to guide occupational therapists who engage in vocational rehabilitation for mental health service users in Namibia. A total of 50 items under seven themes were presented to experts. Overall consensus was reached in 38 items, 25 in the first round and a further 13 in the second round. These included strategies that promote occupational justice, such as advocating for equitable work opportunities, engaging families and employers, employing individualized client‐centered approaches like the “place‐and‐train” model, and aligning practice with national mental health and disability policies. Overall, the findings emphasize the importance of rights‐based, contextually responsive, and collaborative approaches to vocational rehabilitation of mental health service users. This section discusses these findings using an occupational justice lens.

### 4.2. Interpretation of Consensus and Divergence

Out of 50 items presented to experts, 25 items reached consensus in the first round, with an additional 13 items in the second round. Total consensus of 38 out of 50 items represented 76% collective agreement. There were notable shifts between the two Delphi rounds, suggesting participant openness to reconsidering items after reflections. Several items strongly supported by participants align with promoting work as an occupational right of mental health service users [16]. In particular, Items 14–16 and 44 and 45 focuses on advocacy for the right to equitable work, inclusion regardless of illness severity, fair wages commensurate with work done, viewing vocational rehabilitation as a human rights issue, and ensuring vocational rehabilitation is client‐centered, holistic, and culturally sensitive. This finding points to a strong commitment to a justice‐based vocational rehabilitation practice [17]. More so, there is a clear alignment with the occupational justice principle that every person, regardless of disability, has the right to access, engage in, and benefit from meaningful work [18].

Occupational justice stresses the importance of occupational therapists to recognize and dismantle systemic and institutional barriers to participation in occupations, including work [19]. Indeed, six items that participants reached consensus on focus on addressing structural barriers to work participation for mental health services users. These are Item 2, educating employers; Item 3, educating policymakers; Item 4, educating hospital management; Item 6, engaging multidisciplinary teams, and Items 37 and 38, using national disability and mental health policies. By reaching consensus on these items, the participants affirmed the occupational therapists′ role in influencing employment policies, workplace cultures, and health system priorities [20]. This finding supports the notion proposed by Chichaya et al. [21] of reviewing policies and procedures to prevent occupational inconsideration. In their study on analysis of disability policy in Namibia using an occupational justice framework, Chichaya et al. defined occupational inconsideration as a form of occupational injustice that arises when policymakers consciously or unconsciously fail to acknowledge the focal point of occupation in the formulation of disability‐related policies [21]. Therefore, an occupational justice–informed vocational rehabilitation practice framework shall ensure social and structural transformation through addressing structural barriers to work participation for service users [22].

Another key component of occupational justice emerging from these findings is the requirement for interventions to be inclusive, contextually responsive, and capacity building [23]. Items 21–24, 29, 31, 33 and 34 are strategies that focus on place‐and‐train model, negotiating reasonable accommodation, work visits and employer engagement, job matching, personal coaching and life skills training, systematic inclusion of family, and community‐based vocational rehabilitation, respectively. As such, the proposed practice framework shall uphold occupational enablement by ensuring interventions are tailored to service users′ environment and aspirations [23].

Building personal and community capacity to sustain occupational engagement over time is supported by items 13, 25, 26, 28, 33,43, 47 and 50. These highlight self‐employment training, vocational guidance, empowering families, self‐management and treatment adherence, and follow‐up and individualized support. It is imperative that the proposed framework focuses on “rehabilitation through work and support” instead of “rehabilitation to work” [24]. This reframing acknowledges that meaningful occupational engagement is not limited to formal employment alone but includes a spectrum of productive and life‐affirming occupations that contribute to well‐being. By adopting this broader, more inclusive perspective, the proposed framework more fully honors service users′ long‐term potential and dignity, recognizing their capacity to sustain valued roles within their communities over time.

Divergence was noted in the current study. Case in point is Item 18, regular occupational therapy training on vocational rehabilitation; Item 36, setting up employee assistance programs; and Item 40, starting vocational rehabilitation early in hospitalization. Several reasons could account for this divergence. First, the divergence may represent ongoing occupational injustice possibly because of structural or social conditions that limit action [10]. Example could be underresourced vocational rehabilitation structures leading to role strain for occupational therapists who cannot provide optimal service. Consequently, mental health service users experience occupational deprivation [25]. Second, the experts′ understanding of the context could have influenced this finding. Although all the experts were from southern Africa, their vocational rehabilitation experience could have been different from the Namibian situation. Furthermore, hesitance by participants to include traditional leaders, Item 5, could point to discomfort engaging with non‐Western or nonclinical power structures. This is rather unfortunate as traditional healers often shape community norms about inclusion and work for mental health service users.

Given the central role that traditional healers play in providing mental health care across much of sub‐Saharan Africa [26], their limited representation within the expert panel constitutes a notable limitation. The inclusion of a broader range of stakeholders, such as traditional leaders, traditional healers, and community representatives, could have enhanced the cultural and contextual diversity of perspectives informing the study. However, as the proposed framework is primarily intended for hospital‐based intervention, the composition of the panel was aligned with this focus. Future research should prioritize the inclusion of traditional healers, traditional leaders, community representatives, and other community‐based stakeholders to ensure that a wider range of cultural and contextual perspectives are incorporated into the development and refinement of vocational rehabilitation interventions. Such engagement may strengthen the relevance, acceptability, and applicability of these interventions in community‐based settings.

### 4.3. Methodological Reflections

Two Delphi rounds utilized in the present study are methodologically sound and provided a practical balance between achieving meaningful consensus and minimizing fatigue and dropout [8]. The minimal differences observed between the first and second rounds indicate a high level of stability in expert opinion, which is widely recognized as an accepted stopping criterion in Delphi methodology. The second round not only demonstrated increased consensus but also yielded richer qualitative feedback, further strengthening the depth and credibility of the final outcomes. The consistency of responses suggests that participants shared a strong foundational understanding of the contextual realities of occupational therapy practice in southern Africa. This shared professional and regional familiarity may have contributed to early convergence of views, resulting in limited change across rounds while still supporting the robustness of the consensus reached.

The dropout rate was 27% (i.e., from 11 to eight participants), which is within the expected dropout range of 10%–40% [8]. Dropout is not uncommon in Delphi studies; however, in the present study, this could have influenced consensus in second round which was reached with a slightly smaller pool of participants. Furthermore, attrition may have affected the breadth and diversity of perspectives represented, particularly if the views of those who withdrew differed from those who completed all rounds. Consequently, the findings should be interpreted with caution, as the reduced participant pool may have limited the range of expert opinions informing the final consensus. Nevertheless, the panel retained representation from key stakeholder groups, supporting the overall credibility of the study outcomes.

The 3‐point Likert‐type scale utilized offered a simple, intuitive, and low‐burden approach [27]. This was quite important considering that a total of 50 items had to be rated. However, the scale had its limitations. There was a limited discriminatory power and possible ambiguity between *important* and *very important*, resulting in borderline consensus as with Items 2, 25, and 26 [28].

### 4.4. Implications for Practice, Policy, and Research

The study has important implications for occupational therapy practice, policy, and research. In terms of practice, this study affirms the role of occupational therapists as champions of occupational justice by ensuring equitable access to meaningful work opportunities for mental health service users [18]. Supported employment, self‐employment, and job matching are examples of flexible and inclusive vocational rehabilitation approaches that uphold service users′ rights to participation in the occupation of work. Vocational rehabilitation policies should address systematic barriers and promote inclusive and supportive environments to empower work participation for service users [14]. In terms of research, these findings certainly inform the structure of the proposed vocational rehabilitation practice framework. Advocacy, collaboration, rights‐based, and systemic engagement appear to be essential pillars for the framework.

## 5. Conclusion

The modified Delphi study identified clear areas of agreement among occupational therapists in southern Africa regarding their contributions to supporting work‐related participation for mental health service users. The findings show that participants view their role as twofold: providing clinical support to enable skill development and workplace readiness and engaging in practices that address contextual or structural barriers influencing service users′ participation. These perspectives reflect the expert panel′s priorities and professional reasoning, underscoring the importance of integrating both therapeutic and contextual considerations into vocational rehabilitation occupational therapy practice in Namibia. Further research is needed to examine how these roles shape daily practice and to identify ways they can be enhanced to better meet the needs of service users across varied contexts.

## Author Contributions

Four authors contributed to this study. The first author, M.C., a Namibian‐based occupational therapy educator, conducted the research as part of the requirements for a doctoral degree. H.B. and F.A. served as secondary supervisors, whereas L.N. was the primary supervisor. All authors were involved in the conceptualization and design of the study. M.C. led the data collection, analysis, and initial drafting of the manuscript, with ongoing guidance and input from the supervisory team throughout the research process.

## Funding

No funding was received for this manuscript.

## Conflicts of Interest

The authors declare no conflicts of interest.

## Data Availability

The data that support the findings of this study are available from the corresponding author upon reasonable request.
